# Exploring the effect of estrogen on *Candida albicans* hyphal cell wall glycans and ergosterol synthesis

**DOI:** 10.3389/fcimb.2022.977157

**Published:** 2022-09-21

**Authors:** Mohammad Tahseen AL Bataineh, Stefano Cacciatore, Mohammad Harb Semreen, Nihar Ranjan Dash, Nelson C. Soares, Xiaolong Zhu, Muath Khairi Mousa, Jasmin Shafarin Abdul Salam, Luiz F. Zerbini, Rima Hajjo, Mawieh Hamad

**Affiliations:** ^1^ College of Medicine and Health Sciences, Department of Genetics and Molecular Biology, Khalifa University of Science and Technology, Abu Dhabi, United Arab Emirates; ^2^ Molecular Biology and Genetics, Center for Biotechnology, Khalifa University of Science and Technology, Abu Dhabi, United Arab Emirates; ^3^ Bioinformatics Unit, International Centre for Genetic Engineering and Biotechnology, Cape Town, South Africa; ^4^ College of Pharmacy, Department of Medical Chemistry, University of Sharjah, Sharjah, United Arab Emirates; ^5^ College of Medicine, University of Sharjah, Abu Dhabi, United Arab Emirates; ^6^ Research Institute for Medical and Health Sciences at the University of Sharjah, Sharjah, United Arab Emirates; ^7^ Beijing Genomics Institute Genomics, Sharjah, United Arab Emirates; ^8^ Department of Medical Laboratory Sciences, College of Health Sciences, University of Sharjah, Sharjah, United Arab Emirates; ^9^ Cancer Genomics Group, International Centre for Genetic Engineering and Biotechnology, Cape Town, South Africa; ^10^ Department of Pharmacy, Faculty of Pharmacy, Al-Zaytoonah University of Jordan, Amman, Jordan; ^11^ Laboratory for Molecular Modeling, Division of Chemical Biology and Medicinal Chemistry, Eshelman School of Pharmacy, The University of North Carolina at Chapel Hill, Chapel Hill, NC, United States; ^12^ Jordan CDC, Amman, Jordan

**Keywords:** *Candida albicans*, estrogen, ergosterol, turanose, vulvovaginal candidiasis (VC)

## Abstract

Increased levels of 17-β estradiol (E2) due to pregnancy in young women or to hormonal replacement therapy in postmenopausal women have long been associated with an increased risk of yeast infections. Nevertheless, the effect underlying the role of E2 in *Candida albicans* infections is not well understood. To address this issue, functional, transcriptomic, and metabolomic analyses were performed on *C. albicans* cells subjected to temperature and serum induction in the presence or absence of E2. Increased filament formation was observed in E2 treated cells. Surprisingly, cells treated with a combination of E2 and serum showed decreased filament formation. Furthermore, the transcriptomic analysis revealed that serum and E2 treatment is associated with downregulated expression of genes involved in filamentation, including *HWP1*, *ECE1*, *IHD1*, *MEP1*, *SOD5*, and *ALS3*, in comparison with cells treated with serum or estrogen alone. Moreover, glucose transporter genes *HGT20* and *GCV2* were downregulated in cells receiving both serum and E2. Functional pathway enrichment analysis of the differentially expressed genes (DEGs) suggested major involvement of E2 signaling in several metabolic pathways and the biosynthesis of secondary metabolites. The metabolomic analysis determined differential secretion of 36 metabolites based on the different treatments’ conditions, including structural carbohydrates and fatty acids important for hyphal cell wall formation such as arabinonic acid, organicsugar acids, oleic acid, octadecanoic acid, 2-keto-D-gluconic acid, palmitic acid, and steriacstearic acid with an intriguing negative correlation between D-turanose and ergosterol under E2 treatment. In conclusion, these findings suggest that E2 signaling impacts the expression of several genes and the secretion of several metabolites that help regulate *C. albicans* morphogenesis and virulence.

## Introduction

*Candida albicans*, a diploid dimorphic fungus, is humans’ most common opportunistic fungal pathogen, causing mucosal and widespread systemic infections. It is also the most common cause of vaginal candidiasis (VC) in women of childbearing age (Cassone, 2015). Being part of the normal flora in the oral cavity and the gastrointestinal and genitourinary tracts, *C. albicans* is considered a commensal microorganism in healthy individuals. However, *C. albicans* has no known reservoirs outside the mammalian host, and it possesses multiple virulence properties that allow it to cause disease. A major virulence property is the ability to undergo a dimorphic shift from single oval-shaped cells (yeast) into a multicellular form composed of elongated cells attached end-to-end (pseudohyphal and hyphal filaments) (Carlisle et al., 2009; Albataineh et al., 2014; Bataineh MT et al., 2021). Hyphal filaments are associated with virulence and various virulence-related properties, including tissue invasion, lysis of macrophages and neutrophils, and breaching endothelial barriers.

Elevated levels of E2 due to pregnancy or hormonal contraceptives in young women or hormonal replacement therapy in postmenopausal women are associated with an increased risk of VC (Spinillo et al., 1995; Cotch et al., 1998; Cheng et al., 2006). Previous research work suggested that high E2 levels help initiate and maintain VC by affecting the host’s fungus and reproductive tract epithelium (Ferrer, 2000). E2 is also known to exert significant immunosuppressive effects that render the host susceptible to mucosal fungal infections, including VC (Wagner and Johnson, 2012; Hamad, 2012). These effects combined are best illustrated by the fact that E2 is commonly used to induce and maintain experimental VC in animal models (Hirata et al., 2005). The effect of E2 on *C. albicans* was initially studied by measuring its effect on germ tube development and length and culture biomass (White and Larsen, 1997; Gujjar et al., 1997). Biochemical and molecular research later revealed that the localization of the E2 binding protein 1 (Ebp1p) (Skowronski and Feldman, 1989) receptor and the expression of CDR1 and CDR2 in *C. albicans* are influenced by the host E2 levels (Zhang et al., 2000). While E2 was shown to affect *C. albicans* growth, morphology, and antifungal drug responsiveness (Micheli et al., 2002), little is known about its role in the yeast-hyphal transition or the complex regulatory gene expression circuits governing *C. albicans* morphogenesis. Hence, an integrated global view of the cellular and molecular changes E2 treatment induces in *C. albicans* is still needed to develop a better understanding of the role of E2 in *C. albicans* metabolism, morphogenesis and virulence.

Herein, an integrated omics approach, utilizing transcriptomic and metabolomics analyses coupled with functional studies, was employed to better understand the role of E2 in candida filamentation. Similar approaches were previously reported to help in the identification of multiple diagnostic, prognostic, and therapeutic biomarkers and targets in different human diseases (Cavill et al., 2016). In this study, transcriptomic and metabolomic data were integrated with functional data obtained from C*. albicans* cells subjected to temperature and serum induction in the presence or absence of E2 as means of gaining further insight into the role of E2 in *C. albicans growth*, morphogenesis, and virulence.

## Materials and methods

### Strains and growth conditions

As previously described, *C. albicans* wild-type strain (DK318) was used for this study (Banerjee et al., 2008). A Yeast extract-peptone-dextrose (YEPD) medium at 30°C was used as a standard non-filament-inducing growth condition. Serum and temperature liquid induction experiments were performed by growing DK318 overnight in a YEPD medium at 30°C to an optical density at 600 nm (OD_600_) of ∼4.0 and diluting 1:10 into 50 ml of pre-warmed YEPD medium plus 10% serum at 37°C and or 10^-5^ M 17-β-estradiol (E2) as described previously. Aliquots of cells were harvested at specific post-induction time points for RNA preparation.

### RNA preparation and sequencing

RNA extraction was done using RNeasy Micro Kit (Qiagen Ltd. GmbH, Germany) according to the manufacturer’s instructions (Qiagen Ltd.). Three biological replicates were obtained for each treatment condition. In addition, RNA sequencing was performed at BGI Group, Shenzhen, China.

### Data summary

The data set consisted of four candida samples belonging to the DK318 strain under different treatment conditions as follows: DK318 growing in YEPD medium at 30°C (WC) control, DK318 growing in YEPD medium + 10% serum at 37°C (WS), DK318 growing in YEPD medium + E2 at 37°C (WE), and DK318 growing in YEPD medium 10% serum + E2 at 37°C (WES).

### RNA sequencing and filtering

4 samples were sequenced using the BGISEQ-500 platform (Shenzhen, China), generating about 23.97 M reads per sample as previously described (Bataineh MT et al., 2021). After sequencing, reads were filtered using internal software SOAPnuke. The remaining reads were deemed “Clean Reads” and stored in FASTQ format for each sample. Raw data composition filtering statistics and the quality metrics of clean reads are shown in **Supplementary Table S1**.

### Genome mapping

Clean reads were mapped after filtering against the reference genome using HISAT (Hierarchical Indexing for Spliced Alignment of Transcripts) (Bataineh MT et al., 2021). Then, reads were mapped to the *C. albicans* strain SC5314 reference genome (assembly 21) (http://www.candidagenome.org). On average, 97.00% of reads were mapped, and the uniformity of the mapping result for each sample suggested that the samples were comparable. The mapping details (http://www.candidagenome.org ) are shown in **Supplementary Table S2**.

### Gene expression analysis

Clean reads were mapped to reference transcripts using Bowtie2 (Langmead and Salzberg, 2012) v2.2.5, and gene expression levels for genes in each sample were calculated with RSEM v1.2.12. RSEM is a software package that is commonly used to estimate gene and isoform expression levels in RNA-Seq data sets. Clean reads were mapped to reference transcripts using Bowtie2 and calculated gene expression levels for each sample with RSEM to conduct alignments and calculate the FPKM value of genes, respectively (Bataineh MT et al., 2021).

### Determination of DEGs

DEGseq algorithms were employed to detect DEGs between samples and groups. PossionDis method based on Poisson distribution was performed as described in Audic S, et (Audic and Claverie, 1997). was used to detect genes showing significant differential expression between treatment samples and controls.

### Hierarchical clustering analysis of DEGs

Hierarchical clustering for DEGs was performed using the R function *pheatmap*. DEGs were classified according, and GO functional enrichment analysis was performed using phyper, a function of R. The false discovery rate (FDR) for each p-value was then calculated; genes with FDR-adjusted P-value (<0.01) and expression fold changes of more than two or less than −2 were considered as differentially expressed.

Significantly expressed genes were defined as those with an FDR <0.001 and at least a 4-fold change. The R package “pheatmap” was used to plot the heatmap figures to show the degree of difference terms with FDR not larger than 0.01 were defined as significantly enriched. DIAMOND was used to map the DEGs to the STRING database for protein-protein interaction network analysis to obtain the interaction between DEG encoded proteins using homology with known proteins. We select the top 100 interaction networks to draw the picture. For the entire interaction result, we provide an input file that can be imported directly into Cytoscape (Audic and Claverie, 1997; Von Mering et al., 2005; Wang et al., 2010) for network analysis.

### Metabolomics analysis

Metabolite extraction and derivatization was conducted as previously described; briefly, a volume of 300μL of the extraction solvent (acetonitrile: water, 1:1 v/v) was added to the cell pellets (2 million cells per pellet), and the supernatants were collected and transferred to gas chromatography (GC) vials for drying using EZ-2 Plus (GeneVac-Ipswich, UK) at 37 ± 1°C (Bataineh MT et al., 2021). First, the dry samples were dissolved in 25μL of 20 mg/mL methoxyamine hydrochloride in pyridine, followed by the silylation step. Then, for complete derivatization, the samples were incubated at 50°C for 30 min, transferred to 200μL micro-inserts, and analyzed by GC–MS. GC–MS analysis was performed using a QP2010 gas chromatography-mass spectrometer (GC-2010 coupled with a GC–MS QP-2010 Ultra) equipped with an auto-sampler (AOC-20i+s) from Shimadzu (Tokyo, Japan). Chemstation software (Shimadzu). In addition, GC total ion chromatograms (TIC) and fragmentation patterns of the compound were identified using the NIST/EPA/NIH Mass Spectral Library (NIST 14).

### Preprocessing gas chromatography-mass spectrometry data

Preprocessing of metabolomic data was performed using an in-house R script. Probabilistic Quotient Normalization was performed to normalize data due to dilution effects in the extraction procedure using the function normalization in the R package KODAMA (Dieterle et al., 2006; Cacciatore et al., 2017). The number of missing metabolites in the 3 replicates of each condition (i.e., drug and cell line) was counted. When the number was equal to 3, missing values were imputed with zero; otherwise, missing values were imputed using the k-nearest neighbor (kNN) algorithm, with k = 2. By limiting the kNN imputation to the metabolites with at least two values out of 3/condition, imputation using the information from different conditions (e.g., treated and non-treated) was avoided (Troyanskaya et al., 2001). Metabolomic data were mean-centered and scaled to unit variance before being visualized by Principal component analysis (PCA). Two-way ANOVA was used to analyze the filament-inducing (10% fetal bovine serum at 37°C) and E2 treatment effect and to investigate their interaction. The threshold for significance was *p < 0.05*.* A* false discovery rate (FDR) was calculated using the q-value conversion algorithm in multiple comparisons to account for multiple testing. Pathway enrichment analyses were performed using MetaboAnalyst (https://www.metaboanalyst.ca) search against yeast database.

## Results

### E2 signaling is important for *C. albicans* filament extension, but it reduces filamentation in the presence of serum at 37°C.

**Figure 1:**
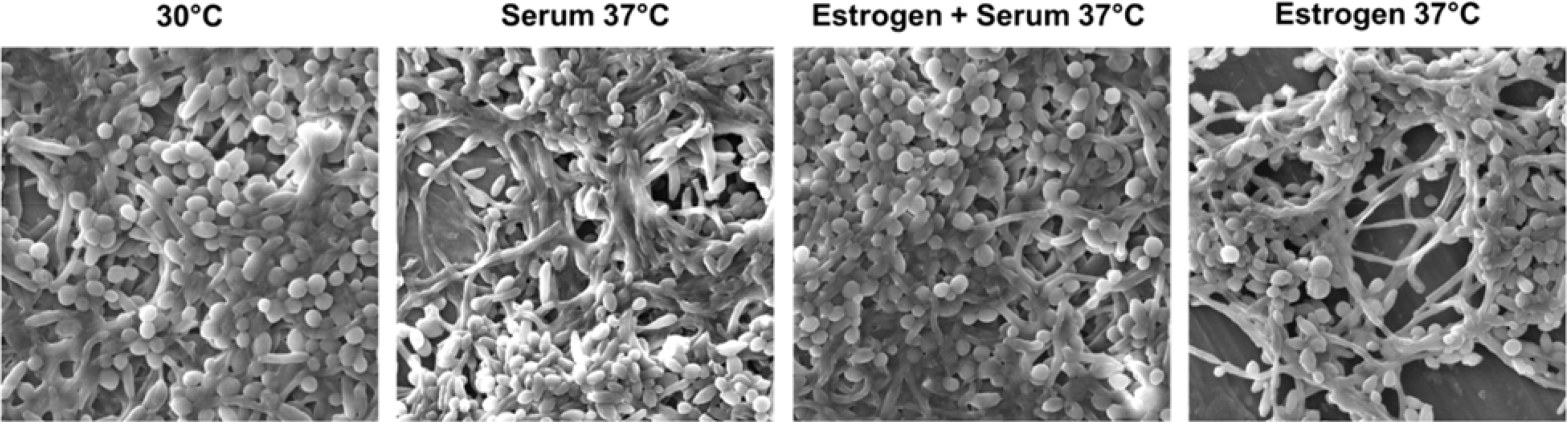
E2 is important for filamentation but reduces filamentation in the presence of serum. Wild-type *C. albicans* strain was grown overnight in YEPD medium at 30°C (non-filament-inducing conditions, 0 hr. time point) and diluted 1:10 into pre-warmed YEPD medium plus 10% serum at 37°C (strong filament-inducing conditions). Cells were harvested at a 3-hour time point, fixed using 4.5% formaldehyde, and washed twice with 1× phosphate-buffered saline (PBS). Images were taken using a scanning electron microscope at 3.00 kx magnification and 15.0 kV acceleration voltage, as shown in the image.

*C. albicans* is the major cause of VC. Previous literature suggests a stimulatory effect of E2 exposure on candida germ tube formation and filamentation (Zhang et al., 2000). In this study, we investigated the impact of 17-β-estradiol on *C. albicans* under different filament-inducing conditions. We observed increased filamentation when candida cells were treated with either serum or E2 alone. However, filamentation was less when cells were treated with both serum and E2 together (**Figure 1**)

Next, we explored the transcriptomic changes associated with *C. albicans* growing under filament-inducing conditions that govern the observed filamentation phenotype in **Figure 1**. RNA sequencing analysis identified a total number of XYZ genes, of which the 435 genes were upregulated genes comparing WS3 *vs*. WE3, whereas only 276 genes were upregulated genes comparing WS3 *vs*. WES3 (276). On the other side, the number of down-regulated genes was higher comparing WS3 *vs*. WES3 (152) than WS3 *vs*. WE3 (133), all compared against WC3 control (**Figure S1**).

### Overall transcriptomic changes of *C. albicans* growing under filament-inducing conditions in response to treatment with E2 at 3 hrs. post-induction

**Figure 2:**
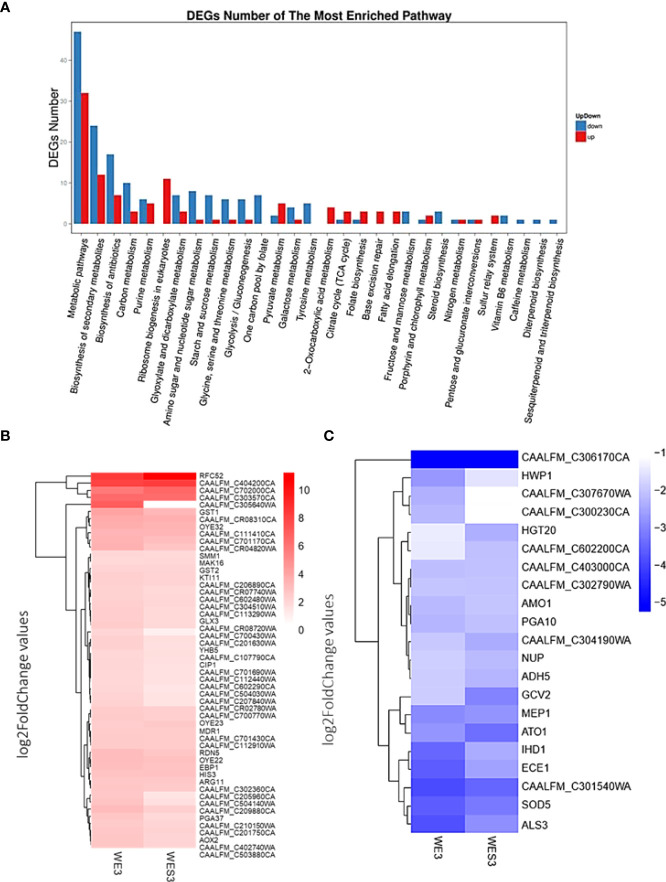
Most enriched GO terms for biological processes and functional enrichment of DEGs in *C. albicans* growing under Serum and Serum with E2 at 3hrs. **(A)** Bar graph representation of significantly up/down-regulated genes, X-axis represents GO term. Y-axis represents the amount of resented GO terms for biological processes in RNA sequencing analysis in response to filament-induction at 3hrs. time point. **(B)** The top 50 upregulated genes and overrepresented GO terms for biological processes upregulated in response to treatment with E2. **(C)** The top 50 down-regulated genes and overrepresented GO terms for biological processes are down-regulated in response to treatment with E2.

To further explore these genes involved in various biological processes, we analyzed the most enriched GO terms for biological processes and functional enrichment of DEGs under serum and serum with E2. In addition, we determined the major involvement of these genes in metabolic pathways and biosynthesis of secondary metabolites (**Figure 2A**).

### Most enriched GO terms for biological processes and functional enrichment of DEGs in *C. albicans* growing under Serum and Serum with E2

**Figure 3:**
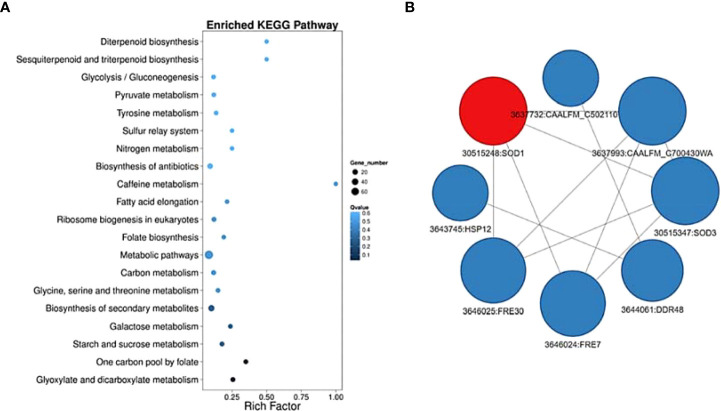
Pathway functional enrichment of DEGs and predicted protein-protein interaction network in *C. albicans* growing under Serum and Serum with E2 at 3hrs. time point. X-axis represents the enrichment factor. Y-axis represents the pathway name. The color indicates the q-value (high: white, low: blue), and the lower q-value indicates the more significant enrichment. Point size indicates DEG number (The bigger dots refer to larger amount). **(A)** Rich Factor refers to the value of enrichment factor, which is the quotient of foreground value (the number of DEGs) and background value (total Gene amount). The larger the value, the more significant enrichment. **(B)** Using STRING database to analyze the protein interaction and construct a visualization of the complex networks, integrating the interaction networks of DEGs. We select the top interaction networks to draw the picture. The red dots refer to upregulated genes in the protein-protein interaction network, while the blue dots refer to down-regulated genes. The size of the circle indicates the number of interactions.

To further describe these genes, we identified the top 50 upregulated genes (**Figure 2B**) and top 50 down-regulated genes (**Figure 2C**) as overrepresented GO terms for biological processes in response to treatment with E2. For example, *HWP1*, *ECE1*, *IHD1*, *MEP1*, *SOD5*, and *ALS3*, all known genes to be important for filamentation, were downregulated comparing WE3 *vs*. WES3 and consistent with the ‘E2 + Serum 37°C’ phenotype observed in **Figure 1**. Furthermore, *HGT20*, a *C. albicans* glucose transporter, and *GCV2*, a glycine decarboxylase important for glycine metabolism in Candida, were also downregulated comparing WE3 *vs*. WES3 (**Figure 2C**). We further verified the pathway functional enrichment of DEGs and predicted protein-protein interaction networks in *C. albicans* growing under Serum and Serum with E2 (**Figure 3**).

### Pathway functional enrichment of DEGs and predicted protein-protein interaction network in *C. albicans* growing under Serum and Serum with E2 at 3hrs. time point.

The results show enrichment of metabolic pathways and interaction between *FRE7* and *FRE30*, ferric reductase enzymes with the *SOD1* and *SOD3*, cytosolic copper-, zinc-, and manganese-containing superoxide dismutase enzymes that protect against oxidative stress and are important for candida virulence (**Figure 3B**). These results prompted us to undergo a metabolic approach to explore these findings further. We conducted a GC-MS analysis of the candida cells growing under the same filament-inducing condition as mentioned before and determined 38 metabolites with varying concentrations between the different treatment conditions (**Supplementary Table S3**).

Among the identified metabolites, we highlight the presence of prominent sugars, alcohols, and organic acids related to energetic metabolisms, such as D-galactose, D-mannitol, D-glycerol, xylitol, and galacturonic acid, glyceric acid, lactic acid (see **Supplementary Table S3**). Additionally, the GC-MS analysis reported many structural carbohydrates and related acids, including xylose, L-Rhamnose, arabinonic acid. Besides sugar and organic acids, the list of detected metabolites was well represented by important fatty acids such as oleic acid, octadecanoic acid,2-keto-D-gluconic acid, palmitic acid, stearic acid. Finally, other compounds such as D-turanose and ergosterol deserved further discussion, as indicated below.

### Metabolic changes in *C. albicans* in control and under filament-inducing conditions

**Figure 4:**
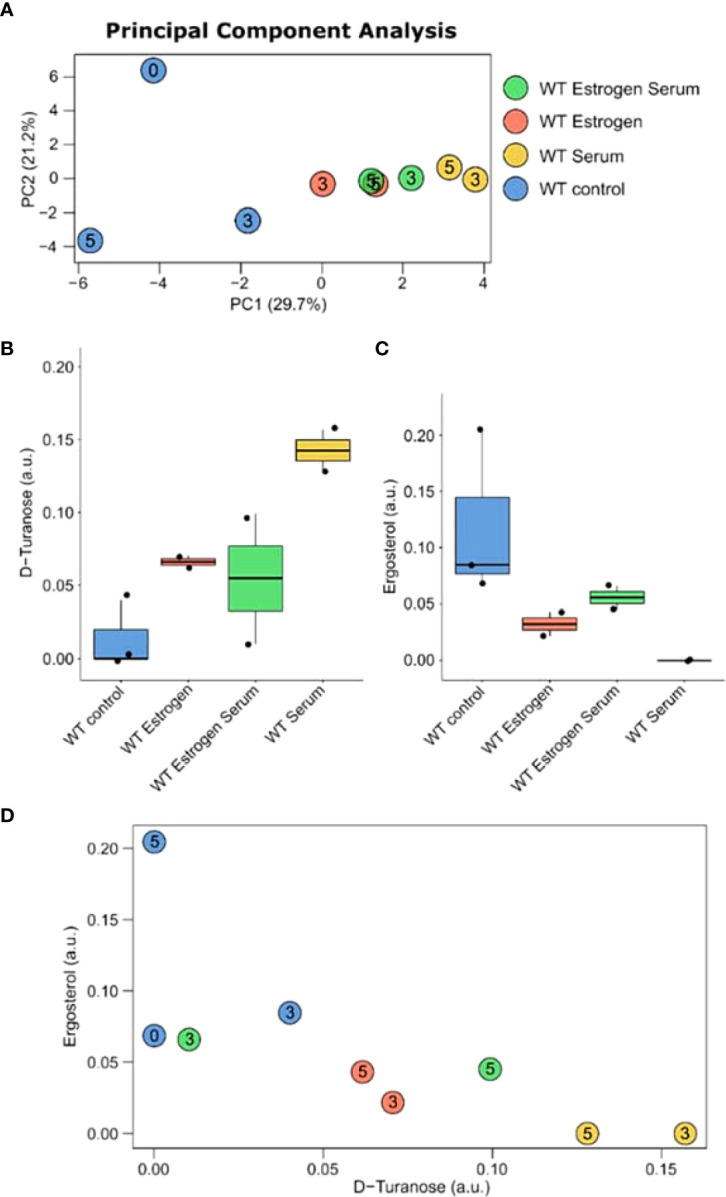
Metabolic changes in **(C)** albicans in control and under filament-inducing (10% fetal bovine serum at 37°C) and/or E2 treatment at different time points (i.e., 0, 3, and 5 hours). **(A)** PCA plot of the metabolic profiles at 0, 3 and 5 hours. Box-and-whisker plots of **(B)** D-turanose and **(C)** ergosterol. **(D)** Correlation plot between the concentration of D-turanose and ergosterol. Rho and p-values are computed using Spearman’s correlation test.

PCA shows on the first component a clear separation between control and filament-inducing and E2 treatment (**Figure 4A**).

Using two-way ANOVA (**Supplementary Table S3**), an interaction effect between E2 and serum treatment for D-turanose (p-value=0.00087) and ergosterol (p-value= 0.0093) was identified (**Figures 4B, C**). A negative correlation was also observed between D-turanose and ergosterol, showing that D-turanose concentration is minimal in cells where ergosterol levels were high (**Figure 4D**). The apparent diversity among the metabolite profiles of *C. albicans* was further confirmed by the pathway enrichment analysis results which prioritized pentose and glucoronate interconversion pathway, glycerolipid metabolism, galactose metabolism, glyoxylate and dicarboxylate metabolism as well as the biosynthesis of unsaturated fatty acids (**Supplementary Figure S2**).

## Discussion

*Candida albicans* is a successful opportunistic microorganism that utilizes several virulence mechanisms that are tightly regulated to allow for efficient invasion, filamentation, and biofilm formation. Additionally, several metabolism-dependent and coordinately-regulated factors are directly or indirectly involved in hyphal cell wall formation, hyphal tip growth, and overall pathogenicity. While global regulation of virulence among fungal pathogens has not been thoroughly investigated, a complex regulatory circuit that involves responses to host-environmental cues such as E2 should be anticipated and evident by the clinical observation of VC.

In this study, we conducted serum and temperature induction experiments in the presence or absence of E2. We noticed a reduction in filamentation when the candida cells were treated with serum plus E2 as opposed to increased filamentation when cells were treated with either serum or E2, separately. Although, we did not conduct a quantitative analysis to calculate yeast and hyphal cells. However, it was clear to us *via* visual examination of 3–4 biological replicates that there was a clear phenotypic difference in filamentation. A negative feedback loop in the presence of both treatments may be responsible for this intriguing finding. Based on this observation, we subsequently sought to explore the global transcriptomic regulation under serum plus E2; a similar analysis was performed on cells subjected to serum or E2 treatment alone as controls. More down-regulated genes (152) were observed when comparing WS3 to WES3 as opposed to comparing WS3 *vs*. WE3 (133) (**Supplementary Figure S1**). To further explore the biological relevance of the gene sets identified in both cases, we analyzed the most enriched GO terms for biological processes and functional enrichment of DEGs under serum alone *vs.* serum plus E2. This analysis showed that several genes involved in hyphal filamentation, including *HWP1*, *ECE1*, and *ALS3*, were significantly downregulated when comparing WE3 to WES3, perhaps suggesting a negative downstream effect of E2 signaling. *HGT20* (a *C. albicans* glucose transporter) and *GCV2* (a glycine decarboxylase important for glycine metabolism in Candida) were also downregulated when comparing WE3 to WES3. Previous studies have elaborated on the crosstalk between sugar signaling and host-environmental cues important for Candida’s filament extension (Le et al., 2010; Albataineh and Kadosh, 2015). In line with this reasoning, it is possible that E2 may affect sugar signaling and auxin responses in an *HGT20*- and *GCV2*-dependant manner. In support of this possibility, our analysis has shown that these same genes are intricately involved in various metabolic pathways and the biosynthesis of multiple secondary metabolites. To further test this hypothesis, metabolomic profiling of *C. albicans* cells treated the same filament-inducing conditions we conducted. No less than 36 metabolic differences between serum alone and serum plus E2 treatments were observed (**Supplementary Table S3**). Interestingly, the levels of D-turanose, nonmetabolizable sucrose, which was previously reported to trigger auxin response (Gonzali et al., 2005), were highly elevated in the presence of E2 or serum and highly reduced in cells showing increased levels of ergosterol. It is possible, therefore, that E2 in *HGT20*- and *GCV2*-dependant mechanisms may regulate the metabolism of D-turanose as a key precursor for glycan synthesis that is important in *C. albicans* filament extension. The negative correlation between turanose and ergosterol, a well-documented player in maintaining candida cell membrane integrity, and the key antimycotic sterol target cannot be readily explained and requires further investigation.

It is well established that serum contains different steroids with different activity and affinity profiles for membrane receptors (Wang et al., 2001). It is also well documented that E2 has high affinities for steroid receptors and can bind to different molecules along the ergosterol biosynthetic pathway (Lv et al., 2016). Moreover, our predicted protein-protein interaction network model shows the interaction between *FRE7* and *FRE30*, ferric reductase enzymes with the *SOD1* and *SOD3*, cytosolic copper-, zinc-, and manganese-containing superoxide dismutase enzymes that protect against oxidative stress and are important for Candida’s virulence. This is consistent with previous literature suggesting a protective role for E2 in upregulating the stress response in *C. albicans* (Lv et al., 2016).

In conclusion, E2 is crucial in controlling regulatory circuits involved in *C. albicans* hyphal filamentation, such as HWP1, ECE1, and ALS3. We uncovered an intriguing phenotype of reduced filamentation after treatment with E2 and serum combined. Contrarily, we observed increased filamentation when the candida cells were treated with either E2 or serum alone. Additionally, we identified a metabolomic signature consisting of anticorrelated D-turanose and ergosterol concentrations (**Figure 4D**). We propose that this metabolomic signature could act as a negative feedback loop to suppress filamentation in a bidirectional E2 *HGT20*- and *GCV2*-dependant mechanisms or by targeting the ergosterol biosynthetic pathways. Finally, we recognize the limitation of the study and aim to validate the E2 requirement for virulence and filamentation in a mouse model of systemic candidiasis and other experiments such as immunofluorescence and Western blotting. Further analysis of the role of E2 in epigenetic modification is also warranted. The identified metabolites could serve as quorum-sensing molecules that govern yeast-to-hyphal transition and are consistent with clinical observation of VC due to elevated E2 levels. It is hoped that an in-depth understanding of the role of E2 in establishing yeast infections is highly clinically relevant and may lead to the discovery of new antifungal therapeutic strategies.

## Data availability statement

The data presented in the study are deposited in the MetaboLights repository, accession number MTBLS5821.

## Author contributions

Conception and design: MB, MH, ND, MS. Acquisition of data: MB, NS, SC, MS, MM, JS. Processing of specimens and data generation: MB, MM, JS, NS, SC, MH, XZ, RH. Analysis and interpretation of data: MB, ND, MH, NS, SC, LZ, XZ, and RH. Manuscript drafting and editing: MB, MH, ND, MH, NS, SC, MM, JS, LZ, RH, XZ. Final approval of manuscript: MB, MH, ND, MH, NS, SC, MM, JS, LZ, RH, and XZ have full access to all study data and take full responsibility for data integrity and accuracy.

## Acknowledgments

The authors wish to acknowledge the generous support of the Research Institute for Medical and Health Sciences, University of Sharjah UAE. We are grateful to David Kadosh (UT Health, San Antonio, USA) for providing *C. albicans* strains. This work was supported by research grants 1701090226-P/MTA, 1901050144/MH, University of Sharjah, Sharjah, UAE.

## Conflict of interest

The authors declare that the research was conducted in the absence of any commercial or financial relationships that could be construed as a potential conflict of interest.

## Publisher’s note

All claims expressed in this article are solely those of the authors and do not necessarily represent those of their affiliated organizations, or those of the publisher, the editors and the reviewers. Any product that may be evaluated in this article, or claim that may be made by its manufacturer, is not guaranteed or endorsed by the publisher.

## Supplementary material

Supplementary Table 1 Raw data composition

Supplementary Table 2 Samples mapping details

Supplementary Table 3 Metabolite concentrations under different filament-inducing conditions. P-values were calculated using a two-way ANOVA test.

Supplementary Figure 1 Volcano plot of DEGs for C. albicans growing under filament-inducing conditions in response to treatment with E2 at 3 hrs. **(A)** Volcano plot of DEGs for WS3 vs. WE3 **(B)** Volcano plot of DEGs for WS3 vs. WES3**(C)** Volcano plot of DEGs for WC3 vs. WS3 **(D)** Volcano plot of DEGs for WC3 vs. WE3 **(E)** Volcano plot of DEGs for WC3 vs. WES3; X axis represents log2 transformed fold change. Y axis represents -log10 transformed significance. Red points represent upregulated DEGs. Blue points represent down-regulated DEGs. Gray points represent non-DEGs. An absolute cutoff value of log fold change >1 (2-fold change) was used. Adjusted P-value 0.05, FDR < 0.001.

Supplementary Figure 2 Visualizing enriched pathways using 36 metabolites identified in the Candida albicans under the tested conditions. Visualizations were performed using MetaboAnalyst pathway enrichment platform. Nodes are colored according to their –log10(p-value) and sized according to the number of associated metabolites.

## Abbreviations

DEG, Differentially Expressed Genes; GO, Gene Ontology; FDR, False Discovery Rate; PCA, Principal Component Analysis; WT, Wild-Type; GC–MS, Gas chromatography-mass spectrometric; YEPD, Yeast extract-peptone-dextrose; HISAT, Hierarchical Indexing for Spliced Alignment of Transcripts.
