# Supplementary material for: Exploring the effect of estrogen on Candida albicans hyphal cell wall glycans and ergosterol synthesis
Source: Front Cell Infect Microbiol. 2022 Sep 21;12:977157. doi: 10.3389/fcimb.2022.977157 (PMC9578540; doi:10.3389/fcimb.2022.977157)
Supplement: Supplementary Table 1 — Raw data composition [file DataSheet_1.docx]

**Table S1.** Raw data composition

| Sample | Total Raw Reads (M) | Total Clean Reads (M) | Total Clean Bases (Gb) | Clean Reads Q20 (%) | Clean Reads Q30 (%) | Clean Reads Ratio (%) |
| --- | --- | --- | --- | --- | --- | --- |
| WC3 | 24.14 | 24.04 | 1.20 | 98.31 | 90.82 | 99.58 |
| WE3 | 24.14 | 23.96 | 1.20 | 98.19 | 90.35 | 99.28 |
| WES3 | 24.14 | 24.02 | 1.20 | 97.92 | 89.60 | 99.50 |
| WS3 | 24.14 | 24.06 | 1.20 | 98.46 | 91.57 | 99.67 |

**Table S2.** Samples mapping details

| Sample | Total Clean Reads | Total Mapping Ratio | Uniquely Mapping Ratio |
| --- | --- | --- | --- |
| WC3 | 24,036,197 | 96.92% | 91.19% |
| WE3 | 23,964,157 | 96.93% | 91.30% |
| WES3 | 24,015,996 | 97.25% | 91.31% |
| WS3 | 24,058,441 | 97.12% | 91.31% |

**Table S3.** Metabolite concentrations under different filament-inducing conditions. P-values were calculated using a two-way ANOVA test.

|  | Metabolite concentration | | | | E2  treatment | | Serum treatment | | Interaction E2: Serum | |
| --- | --- | --- | --- | --- | --- | --- | --- | --- | --- | --- |
| etabolite | *WT control* | *WT E2* | *WT E2+ Serum* | *WT Serum* | *p-value* | *FDR* | *p-value* | *FDR* | *p-value* | *FDR* |
| Octadecanol | 0.62 1.13 2.02 | 0.73 0.80 | 1.08 0.93 | 0.81 0.62 | 7.17E-01 | 7.98E-01 | 6.04E-01 | 8.36E-01 | 2.35E-01 | 7.17E-01 |
| 2-Aminobenzoic acid | 0.66 0.90 0.92 | 1.18 0.97 | 0.96 0.00 | 0.00 0.00 | 3.02E-01 | 6.83E-01 | 1.95E-02 | 2.34E-01 | 5.13E-01 | 7.17E-01 |
| 2-Keto-d-gluconic acid | 0.33 1.34 0.67 | 0.12 0.72 | 0.00 0.00 | 0.00 0.00 | 3.23E-01 | 6.84E-01 | 3.14E-02 | 2.38E-01 | 5.01E-01 | 7.17E-01 |
| 2,3-Butanediol | 0.00 0.28 0.00 | 8.31 0.00 | 0.00 6.39 | 0.00 0.00 | 1.75E-01 | 6.83E-01 | 8.85E-01 | 9.15E-01 | 9.81E-01 | 9.81E-01 |
| Oleic acid amide | 0.09 0.00 0.00 | 1.42 1.49 | 1.93 0.00 | 3.94 0.44 | 4.17E-01 | 6.97E-01 | 3.54E-01 | 7.07E-01 | 1.20E-01 | 6.68E-01 |
| Acetoin | 0.00 1.48 0.43 | 1.93 0.00 | 0.00 4.09 | 0.00 0.00 | 3.98E-01 | 6.97E-01 | 8.28E-01 | 9.03E-01 | 4.83E-01 | 7.17E-01 |
| Succinic acid | 3.59 1.12 0.28 | 1.74 0.00 | 0.00 0.39 | 0.00 0.00 | 6.50E-01 | 7.98E-01 | 1.44E-01 | 4.00E-01 | 5.14E-01 | 7.17E-01 |
| D-(+)-Galactose | 0.76 0.73 0.88 | 0.00 0.00 | 0.00 1.45 | 0.00 2.34 | 3.04E-01 | 6.83E-01 | 5.31E-01 | 7.97E-01 | 5.38E-01 | 7.17E-01 |
| D-(+)-Turanose | 0.00 0.80 0.00 | 1.41 1.23 | 0.21 1.98 | 3.14 2.56 | 7.41E-01 | 7.98E-01 | 7.98E-02 | 3.42E-01 | 4.02E-02 | 6.68E-01 |
| D-Mannitol | 7.75 0.00 0.00 | 0.00 0.00 | 0.00 0.00 | 0.00 0.00 | 4.51E-01 | 6.97E-01 | 4.93E-01 | 7.72E-01 | 5.30E-01 | 7.17E-01 |
| D-Xylose | 0.16 0.00 0.23 | 0.00 0.00 | 0.32 0.00 | 0.00 0.00 | 9.84E-01 | 9.84E-01 | 9.86E-01 | 9.86E-01 | 1.46E-01 | 6.68E-01 |
| Ergosterol | 1.70 2.09 5.06 | 0.54 1.07 | 1.63 1.12 | 0.00 0.00 | 7.54E-01 | 7.98E-01 | 3.30E-02 | 2.38E-01 | 1.09E-02 | 3.94E-01 |
| Galacturonic acid | 0.00 8.28 0.00 | 0.00 0.00 | 0.00 0.00 | 5.34 0.00 | 2.79E-01 | 6.83E-01 | 8.90E-01 | 9.15E-01 | 8.99E-01 | 9.52E-01 |
| Glyceric acid | 7.46 0.00 6.32 | 0.00 0.00 | 0.00 0.00 | 0.00 0.00 | 1.64E-01 | 6.83E-01 | 2.00E-01 | 5.15E-01 | 2.36E-01 | 7.17E-01 |
| Glycerol monostearate | 1.34 0.77 0.67 | 1.86 0.00 | 0.00 1.53 | 0.00 0.00 | 7.40E-01 | 7.98E-01 | 2.74E-01 | 6.57E-01 | 3.89E-01 | 7.17E-01 |
| Glycerol | 1.96 1.18 3.77 | 1.00 0.03 | 1.00 1.18 | 0.70 1.11 | 1.42E-01 | 6.83E-01 | 6.59E-01 | 8.48E-01 | 1.11E-01 | 6.68E-01 |
| Glycolic acid | 0.00 1.84 1.80 | 2.33 0.00 | 0.00 1.60 | 0.00 0.00 | 7.76E-01 | 7.98E-01 | 3.44E-01 | 7.07E-01 | 5.26E-01 | 7.17E-01 |
| Gulonic acid | 11.46 0.91 0.88 | 0.24 0.00 | 0.00 0.00 | 0.87 0.97 | 9.08E-02 | 6.83E-01 | 4.47E-01 | 7.72E-01 | 6.09E-01 | 7.83E-01 |
| L-Rhamnose | 0.00 0.00 2.87 | 0.00 0.00 | 0.00 0.00 | 4.84 0.00 | 2.68E-01 | 6.83E-01 | 6.60E-01 | 8.48E-01 | 6.87E-01 | 8.49E-01 |
| L-Valine | 1.48 0.13 1.00 | 0.00 0.00 | 0.00 6.36 | 0.00 0.00 | 7.51E-01 | 7.98E-01 | 7.74E-01 | 8.99E-01 | 1.48E-01 | 6.68E-01 |
| Lactic Acid | 3.62 0.42 0.56 | 0.38 0.52 | 0.00 0.25 | 0.00 0.00 | 4.64E-01 | 6.97E-01 | 1.15E-01 | 3.46E-01 | 4.06E-01 | 7.17E-01 |
| Malic acid | 0.40 1.06 1.78 | 2.01 1.66 | 0.00 1.47 | 0.00 0.00 | 2.32E-01 | 6.83E-01 | 4.61E-02 | 2.76E-01 | 8.37E-01 | 9.13E-01 |
| Malonic acid | 0.00 0.00 4.64 | 0.00 0.00 | 0.00 0.00 | 0.00 0.00 | 4.51E-01 | 6.97E-01 | 4.93E-01 | 7.72E-01 | 5.30E-01 | 7.17E-01 |
| Myo-Inositol | 3.05 0.91 0.39 | 0.66 0.85 | 0.87 1.37 | 1.31 1.20 | 5.68E-01 | 7.87E-01 | 7.10E-01 | 8.82E-01 | 7.50E-01 | 8.71E-01 |
| N,N-Dimethylglycine | 2.79 0.63 2.48 | 1.92 0.00 | 0.00 0.88 | 0.00 0.00 | 5.96E-01 | 7.95E-01 | 1.04E-01 | 3.42E-01 | 2.83E-01 | 7.17E-01 |
| Octadecanoic acid | 0.00 0.00 0.00 | 0.00 0.00 | 12.78 0.00 | 0.00 0.00 | 2.92E-01 | 6.83E-01 | 3.35E-01 | 7.07E-01 | 2.96E-01 | 7.17E-01 |
| Palmitic Acid | 1.18 1.94 3.92 | 1.09 1.52 | 0.91 1.46 | 2.60 1.40 | 1.80E-01 | 6.83E-01 | 7.66E-01 | 8.99E-01 | 9.71E-01 | 9.81E-01 |
| Pentitol | 1.03 1.98 7.79 | 2.35 0.00 | 0.00 0.69 | 0.00 0.00 | 4.37E-01 | 6.97E-01 | 9.61E-02 | 3.42E-01 | 3.06E-01 | 7.17E-01 |
| Pipecolic acid | 0.57 3.71 4.13 | 0.84 0.00 | 0.00 0.50 | 0.00 0.00 | 1.99E-01 | 6.83E-01 | 7.93E-02 | 3.42E-01 | 1.46E-01 | 6.68E-01 |
| Pyroglutamic acid | 1.05 0.00 0.00 | 3.80 0.00 | 0.00 3.76 | 0.00 0.00 | 2.59E-01 | 6.83E-01 | 8.03E-01 | 9.03E-01 | 8.25E-01 | 9.13E-01 |
| Ribitol | 1.65 1.59 1.37 | 0.88 1.92 | 0.00 0.00 | 0.00 0.00 | 2.33E-01 | 6.83E-01 | 2.62E-04 | 4.72E-03 | 7.07E-01 | 8.49E-01 |
| Scyllo-Inositol | 3.39 0.00 0.00 | 0.00 0.00 | 0.00 0.00 | 0.00 0.00 | 4.51E-01 | 6.97E-01 | 4.93E-01 | 7.72E-01 | 5.30E-01 | 7.17E-01 |
| Stearic acid | 1.03 11.19 5.60 | 0.00 0.00 | 0.00 0.45 | 0.00 0.00 | 6.53E-02 | 6.83E-01 | 8.66E-02 | 3.42E-01 | 6.40E-02 | 6.68E-01 |
| Tryptophol | 2.15 0.00 1.43 | 0.00 0.00 | 0.00 0.00 | 0.69 0.00 | 1.25E-01 | 6.83E-01 | 4.54E-01 | 7.72E-01 | 4.93E-01 | 7.17E-01 |
| Uridine | 0.60 4.11 0.00 | 0.00 0.00 | 0.00 0.85 | 0.00 0.00 | 5.14E-01 | 7.41E-01 | 5.54E-01 | 7.97E-01 | 2.45E-01 | 7.17E-01 |
| Xylitol | 0.00 0.00 0.00 | 0.00 0.00 | 1.70 1.12 | 2.95 1.68 | 7.22E-01 | 7.98E-01 | 1.26E-04 | 4.55E-03 | 1.76E-01 | 7.03E-01 |


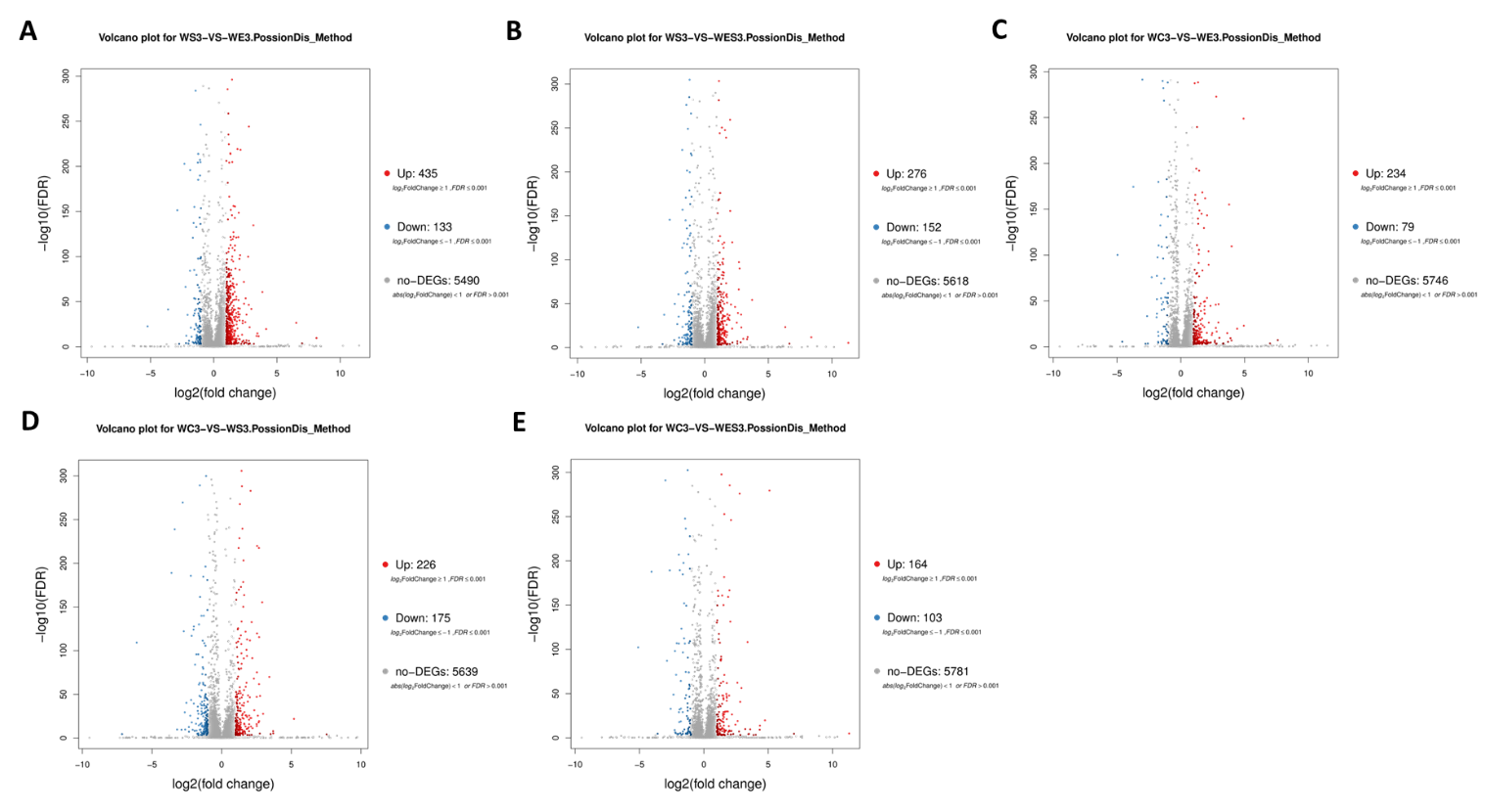


**Figure S1**. Volcano plot of DEGs for *C. albicans* growing under filament-inducing conditions in response to treatment with E2 at 3 hrs. (A) Volcano plot of DEGs for WS3 *vs*. WE3 (B) Volcano plot of DEGs for WS3 vs. WES3(C) Volcano plot of DEGs for WC3 *vs*. WE3 (D) Volcano plot of DEGs for WC3 *vs*. WS3 (E) Volcano plot of DEGs for WC3 *vs*. WES3; X axis represents log2 transformed fold change. Y axis represents -log10 transformed significance. Red points represent upregulated DEGs. Blue points represent down-regulated DEGs. Gray points represent non-DEGs. An absolute cutoff value of log fold change >1 (2-fold change) was used. Adjusted P-value 0.05, FDR <0.001.


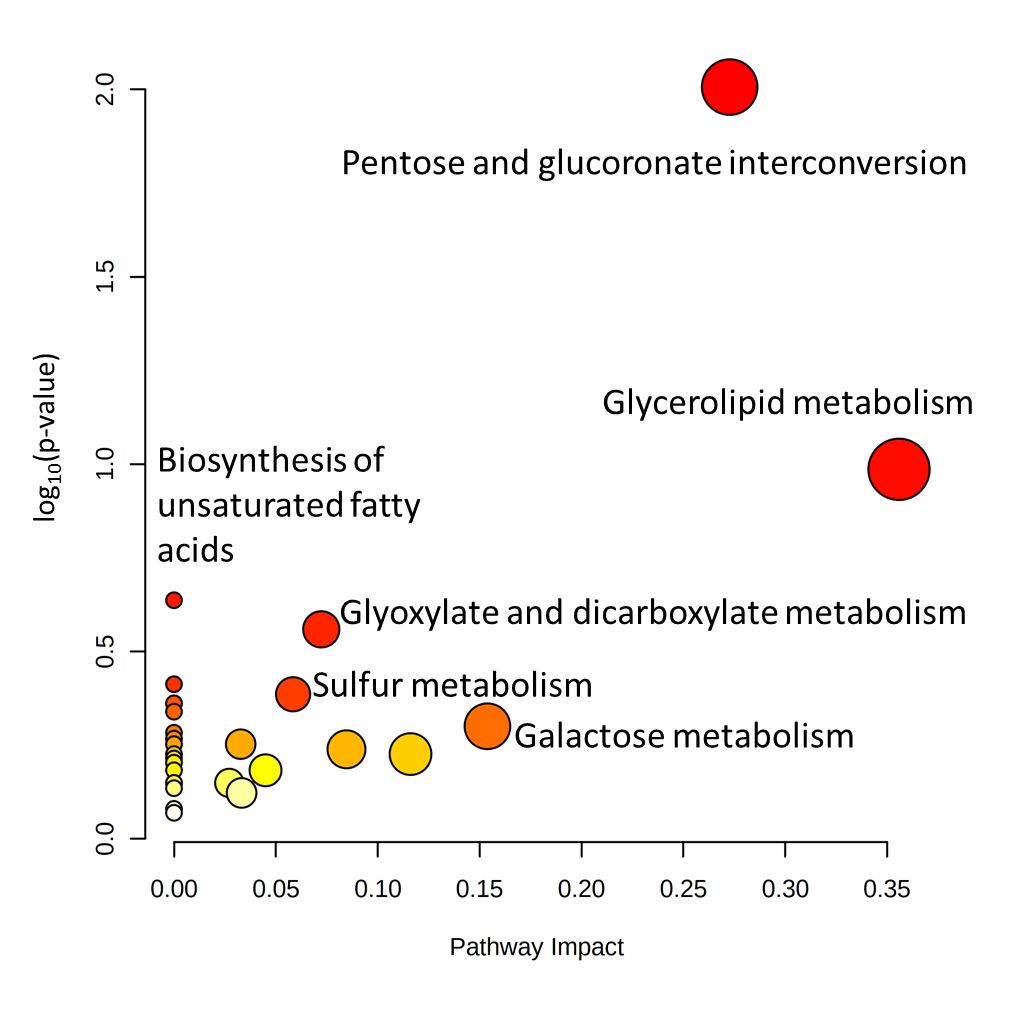


**Figure S2.** Visualizing enriched pathways using 36 metabolites identified in the *Candida albicans* under the tested conditions. Visualizations were performed using MetaboAnalyst pathway enrichment platform. Nodes are colored according to their –log_10_(p-value) and sized according to the number of associated metabolites.
